# The Spectrum of *C9orf72*-mediated Neurodegeneration and Amyotrophic Lateral Sclerosis

**DOI:** 10.1007/s13311-015-0342-1

**Published:** 2015-03-03

**Authors:** Johnathan Cooper-Knock, Janine Kirby, Robin Highley, Pamela J. Shaw

**Affiliations:** Sheffield Institute for Translational Neuroscience, Department of Neuroscience, University of Sheffield, 385A Glossop Road, Sheffield, S10 2HQ UK

**Keywords:** Amyotrophic lateral sclerosis, Frontotemporal dementia, *C9orf72*, RNA foci, Dipeptide repeat proteins, Antisense oligonucleotides

## Abstract

The discovery that a hexanucleotide repeat expansion in *C9orf72* is the most numerous genetic variant of both amyotrophic lateral sclerosis and frontotemporal dementia has opened a rapidly growing field, which may provide long hoped for advances in the understanding and treatment of these devastating diseases. In this review we describe the various phenotypes, clinical and pathological, associated with expansion of *C9orf72*, which go beyond amyotrophic lateral sclerosis and frontotemporal dementia to include neurodegeneration more broadly. Next we take a step back and summarize the current understanding of the *C9orf72* expansion and its protein products at a molecular level. Three mechanisms are prominent: toxicity mediated directly by RNA transcribed from the repeat; toxicity mediated by dipeptide repeat proteins translated from the repeat sequence; and haploinsufficiency resulting from reduced transcription of the *C9orf72* exonic sequence. A series of exciting advances have recently described how dipeptide repeat proteins might interfere with the normal role of the nucleolus in maturation of RNA binding proteins and in production of ribosomes. Importantly, these mechanisms are unlikely to be mutually exclusive. We draw attention to the fact that clinical and pathological similarities to other genetic variants without a repeat expansion must not be overlooked in ascribing a pathogenic mechanism to *C9orf72*-disease. Finally, with a view to impact on patient care, we discuss current practice with respect to genetic screening in patients with and without a family history of disease, and the most promising developments towards therapy that have been reported to date.

**Electronic supplementary material:**

The online version of this article (doi:10.1007/s13311-015-0342-1) contains supplementary material, which is available to authorized users.

Amyotrophic lateral sclerosis (ALS) is a progressive neurodegenerative disease caused by the loss of upper motor neurons in the motor cortex and lower motor neurons in the brainstem and spinal cord. The majority of cases are sporadic (SALS) but 5–10 % are familial (FALS), usually with autosomal dominant inheritance. In 1993, the first pathogenic genetic mutations were identified affecting Cu–Zn superoxide dismutase 1 (SOD1); SOD1 mutations account for between 12 % and 24 % of FALS cases, depending on the population sampled [1]. There are now mutations in > 20 genes identified as causative in ALS [2], of which the most common are mutations in *SOD1*, the RNA binding protein encoding genes *TARDBP* and *FUS,* and the recently identified *C9orf72*. Repeat expansions of *C9orf72* have also been associated with neurodegeneration elsewhere in the central nervous system (CNS), within and outside of the motor system, as will be explored below.

## Identification of the *C9orf72* Repeat Expansion

The *C9orf72* GGGGCC expansion was identified through study of a risk haplotype at the 9p21 locus. Several investigations had identified the locus through linkage analysis in both FALS and SALS, as well as FALS associated with frontotemporal dementia (ALS-FTD) [3–8]. The delay of > 5 years between discovery of the locus and identification of the responsible mutation is largely owing to the fact that the expansion is intronic and has 100 % GC content, which made it difficult to detect by traditional sequencing techniques. Expansions of > 30 repeats were eventually linked to the disease using sequencing based on repeat-primed polymerase chain reaction (PCR) [9, 10]. Association of the *C9orf72* with the same risk haplotype in various populations has led to the proposal that a common founder is responsible for all of cases identified [11], but this is controversial [12–15]. As *C9orf72* disease has a late age of onset the mutation is not subject to reproductive pressure, which helps explain how such a devastating mutation could have become so widespread.

Observations of population frequencies of the expansion are consistent with a common founder effect. In Finland *C9orf72* expansions were found in 61 % of patients with FALS and 19 % of patients with SALS [11]; however, further away from Scandinavia the expansion frequency becomes less. In northern England *C9orf72* expansions are present in 43 % of patients with ALS with an identifiable family history and in 7 % of apparently sporadic cases [16]. In Germany 22 % of patients with FALS carry the expansion [11], but in Japan the equivalent figure is only 3.4 % [17]. In North America the frequency of the *C9orf72* expansion appears to be comparable with European populations: it is reported that 36 % of patients with FALS and 6 % of patients with SALS carry the expansion [11]. As might be expected, there is some variability depending on ethnic group, for example no Native Americans were identified with the expansion, but the numbers screened were small.

It is possible that it is not the expansion itself that is inherited but a propensity for the region to expand. One of the initial studies of *C9orf72* noted that the risk haplotype was associated with an increased number of repeats, even in controls [10]. More recently, it has been shown that a 9p21 haplotype is significantly associated with SALS, even if patients with the GGGGCC repeat expansion are excluded [18]; perhaps an alternative, as yet unidentified, repeat sequence is present at the same locus.

## Clinical Phenotypes Associated with the *C9orf72* Expansion in ALS

The majority of non-*C9orf72* ALS cases are limb onset, while 25 % of patients present with bulbar onset and only 3 % with respiratory onset [19]. Mean survival is 32 months from symptom onset, although 14–24 % of patients have a disease duration of > 5 years and 4 % > 10 years [20]. Generally, there is no overt cognitive dysfunction at disease onset, but as the disease progresses cognitive impairment can develop in up to 50 %, with clinically defined FTD occurring in 13–14 % of cases [21, 22].

In *C9orf72* ALS, while the full spectrum of the ALS clinical phenotypes described above is represented, the most significant and robust clinical feature associated with patients carrying a repeat expansion is the increased incidence of FTD or a family history of FTD in up to 50 % of cases [16, 23–27]. This is not surprising given that the 9p21 locus was initially identified through mapping ALS-FTD families, where cases presented with either ALS or FTD, or both diseases [28, 29]. There is also an increase in the incidence of bulbar onset in *C9orf72* ALS of up to 44 % compared with an average of 25–26 % in non-*C9orf72* ALS [16, 24, 26], and several groups also found evidence of an earlier age of onset by 1.8–5.0 years [23, 25, 27, 30]. *C9orf72* ALS has also been associated with a shorter disease duration by 5.7–12.0 months, suggesting a more aggressive disease course [16, 23, 25, 27].

While not all clinical cohorts show all of these characteristics, the inconsistencies may not only be owing to the different populations under consideration, but also to the groups under comparison, as some reports compare *C9orf72* ALS with all non-*C9orf72* ALS cases, whereas others compare *C9orf72* ALS specifically with familial or sporadic non-*C9orf72* ALS. This is highlighted in a Belgian cohort of patients with ALS, where comparing familial *C9orf72* carriers with non-*C9orf72* FALS cases showed a significant increase in the frequency of bulbar onset, an increase in the age of onset, an increase in cases with a history of FTD, and a reduction in survival in *C9orf72* ALS [31]. However, the clinical phenotype of sporadic *C9orf72* ALS cases was not significantly different from that of non-*C9orf72* SALS. Similarly, Millecamps et al. [32] compared the clinical phenotype of *C9orf72* ALS with that of known SOD1, TAR DNA binding protein 43 (TDP-43) and fused in sarcoma (FUS)-related ALS patients. Again, bulbar onset was found more frequently in *C9orf72* ALS than in the other 3 genetic variants. While *C9orf72* ALS cases had shorter disease duration compared with SOD1 and TDP-43-related ALS cases, and an older age of onset compared with SOD1 and FUS-related ALS, the consistent finding in this analysis was the increased incidence of FTD or a family history of dementia identified in the patients with *C9orf72* ALS. In families where ALS-FTD is the clinical phenotype, mutation screening has shown that the *C9orf72* expansion accounts for around ≥ 50 % of the cases [33–37]. Finally, given the variability in *C9orf72* disease, it is likely that there are a significant number of genetic and environmental modifiers; a number of candidates, including the length of the repeat expansion, will be discussed below.

### ALS and Parkinson’s Disease/Parkinsonism

During the screening of *C9orf72* in ALS cohorts, it was noted that there was also an apparent increase in the incidence of Parkinson’s disease (PD), parkinsonism concomitant with ALS or a family history of PD [16, 33]. However, screening for the *C9orf72* expansion in cohorts of PD cases identified a few rare incidences of the repeat expansion, usually in cases with atypical Parkinson’s disease [38–40]. Given the high incidence of ALS and parkinsonism-dementia-complex in the Kii peninsula of Japan and in the Chamorro inhabitants of Guam, individuals were screened to see if the *C9orf72* expansion contributed to this pathology. Whereas no *C9orf72* expansions were found in the Charmorro population [41], 3 (20 %) ALS cases carried an expansion in the Kii Peninsula, a frequency significantly higher than across the rest of Japan, suggesting *C9orf72* expansions do contribute to the prevalence of ALS in this high incidence geographical focus [42].

Further reports failed to find *C9orf72* expansions over 30 repeats in PD in the Chinese Han and US white populations [43, 44]. However, both disease cohorts showed an increase in the number of intermediate repeats compared with controls. In the American population 14 cases had 20–30 repeats (compared with 3 controls), while 7 cases had > 23 repeats [44]. In the Chinese population, 3 PD cases had repeat lengths of 20–30 (compared with zero controls) and > 7 repeats was found to be significantly associated with PD [43].

## Spectrum of *C9orf72*-associated Diseases

As well as ALS, *C9orf72* GGGGCC repeat expansions are commonly found in FTD, and the spectrum of *C9orf72* diseases extends from both ALS and FTD to other motor disorders such as primary lateral sclerosis (PLS), progressive muscular atrophy (PMA), and Huntington’s disease (HD) phenocopies, as well as other non-motor disorders, such as Alzheimer’s disease (AD).

### Motor Disorders Associated with C9orf72 Repeat Expansions

While ALS is characterized by the degeneration of both the upper and lower motor neurons, PLS is caused by the loss of upper motor neurons and PMA by the loss of lower motor neurons. These are predominantly sporadic disorders. However, screening of *C9orf72* has found repeat expansions to be present within these disease phenotypes, in 0.9–8.7 % of PLS cases and 1.6–25.0 % of PMA cases from Dutch, Canadian, and Spanish populations [24, 27, 30]. In contrast, no expansions were seen in PLS and PMA cohorts from Germany and the USA [37, 45]. They were also absent from cohorts of spinal muscular atrophy and patients with hereditary spastic paraplegia [45–47].

*C9orf72* has also been screened for in HD-like syndromes, where repeat expansions were found in 7 cases, at a frequency of 1.7 %, which was significantly more frequent than the presence of expansions in controls (0.15 %) [14]. Subsequently, in a large cohort of 514 HD phenocopies, expansions were identified in 10 cases (1.95 %), thereby establishing *C9orf72* repeat expansions as the most commonly identified genetic cause of a HD mimic syndrome [48]. In this study, HD phenocopies were identified by the presence of a clinical triad of psychiatric, movement, and cognitive impairment in the absence of a CAG repeat expansion of huntingtin. In all cases diagnosis was made by an experienced clinician, although, of course, when classification is purely clinical there remains the possibility that some of these cases may have been wrongly selected.

Rare cases of corticobasal syndrome have been reported as having a *C9orf72* expansion [39, 40], as has a case of progressive supranuclear palsy [39]. In a cohort of 209 cases of spinocerebellar ataxia, an expansion was found in a single patient, whose father also carried the repeat and had ALS [49]. In addition, a brother and sister, both of whom were carrying a *C9orf72* expansion, presented with ALS and multiple system atrophy, respectively [50]. Finally, a *C9orf72* expansion was also found in a Finnish patient who developed a dysplastic gangliocytoma usually associated with mutations in *PTEN*. This individual also exhibited the characteristic pathology associated with *C9orf72* expansions (see ‘Neuropathology of *C9orf72* Repeat Expansions’). Whether or not these later examples are rare coincidences, or specifically related to the *C9orf72* expansion, remains to be determined. However, it is clear from the PMA, PLS, and HD phenocopies that the *C9orf72* phenotype extends beyond the classical motor disorder of ALS.

### Non-motor Disorders with C9orf72 Repeat Expansions

Following identification of the GGGGCC repeat expansion in *C9orf72* in ALS-FTD, screening of FTD cases found the repeat expansion accounted for 25.1 % of familial FTD and 5.8 % of sporadic cases worldwide, although, as with ALS, the frequency does vary within different populations [11]. While FTD can present as behavioral variant FTD (bvFTD), progressive nonfluent aphasia, or semantic dementia, those with the *C9orf72* expansion predominantly present with bvFTD, including progressive personality deterioration, such that affected individuals may exhibit psychosis in the form of hallucinations and delusions [11]. The frequency of bvFTD is consistently higher in patients with *C9orf72* FTD than in non-*C9orf72* FTD cases in multiple populations across the world [13, 36, 51, 52]. Some patients with *C9orf72* expansions do present with PNFA with loss of word retrieval and nonfluent speech culminating in loss of speech, but the frequency of this presentation was similar in both *C9orf72* and non-*C9orf72* cohorts [36]. In contrast, semantic dementia, where individuals lose their understanding of words and objects, is only rarely associated with *C9orf72* expansion [51, 53].

As *C9orf72* repeat expansions are associated with dementia, cases with AD were screened to determine whether the GGGGCC repeat also contributed to AD. Some cohorts do contain *C9orf72* expansions at relatively low frequencies (0.76 %), but this includes cases with pathologically confirmed AD [54–56]. However, other reports have failed to identify any expansions with > 30 repeats in AD cases [52, 57, 58]. In addition,owing to the prevalence of psychosis symptoms associated with *C9orf72* FTD, cohorts of schizophrenia patients have also been screened for the expansion [59, 60]. However, only 1 report has identified repeats > 30 in 2 of 298 (0.67 %) patients with schizophrenia [61]. Similarly, only 1 individual with bipolar disorder has been found with a *C9orf72* repeat expansion, although the affected parent who passed on the repeat and was originally diagnosed with bipolar disorder went on to also develop FTD [62]. It remains to be established if these mutations are truly causative in these non-motor system disorders or if the associations are due to chance, as *C9orf72* expansions are seen in 0.15 % of UK controls [14], and the expansion has been reported to show incomplete penetrance in an Italian cohort [63].

## Neuropathology of *C9orf72*-related ALS

**Fig. 1:**
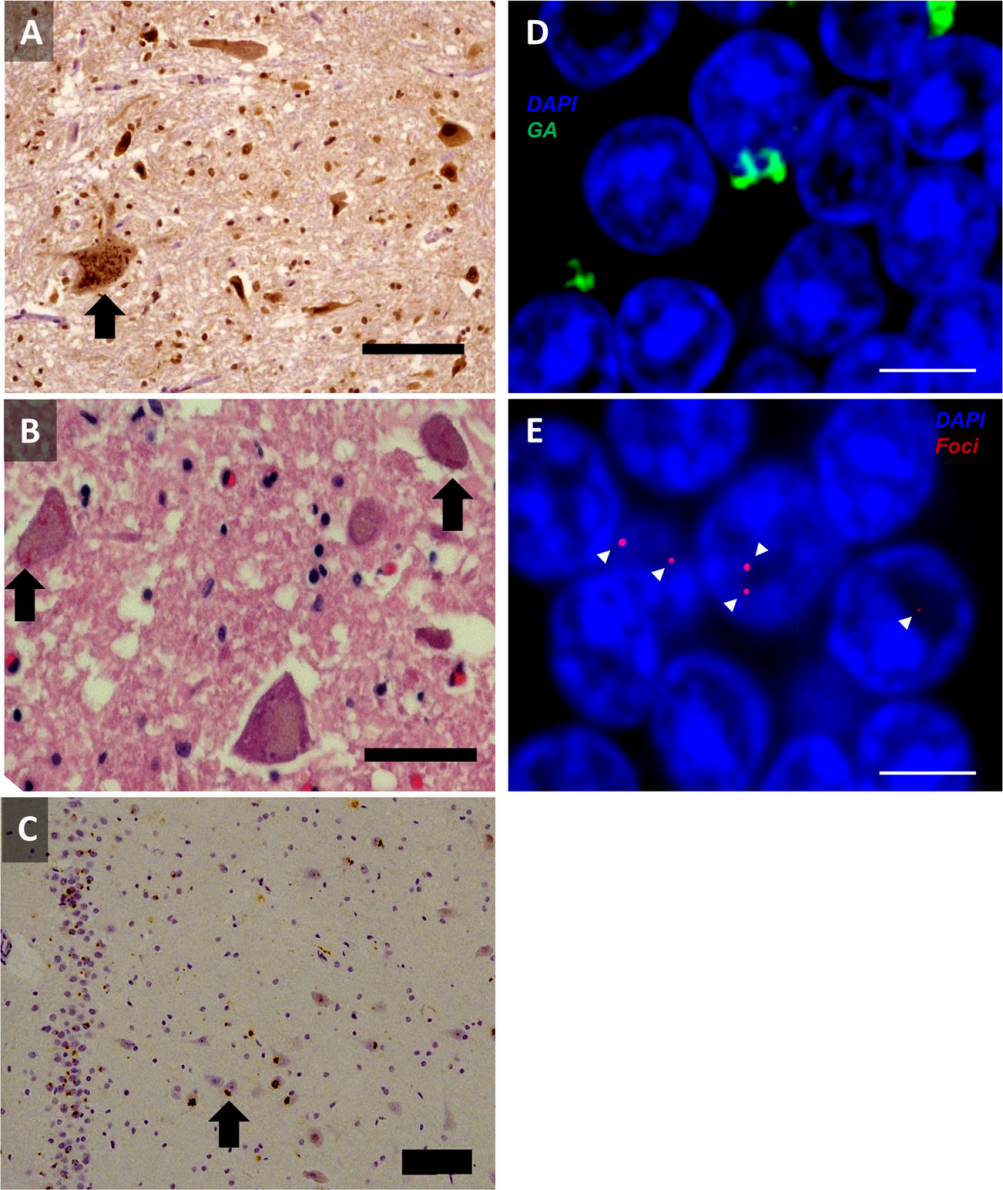
Characteristic pathology of *C9orf72* amyotrophic lateral sclerosis (ALS). Motor neurons of the spinal cord display typical TAR DNA binding protein-43 (TDP-43) pathology, including cytoplasmic TDP-43 positive skeins and compact inclusions [(A), anterior horn, stained with 3,3' diaminobenzidine (DAB) for pTDP-43, scale bar 100 μm] and Bunina bodies [(B), anterior horn, hematoxylin and eosin stain, scale bar 100 μm]. However, in addition, patients with *C9orf72* ALS display p62-positive cytoplasmic inclusions in extramotor areas [(C), hippocampus dentate gyrus, DAB stained for p62, scale bar 100 μm], which also stain for dipeptide repeat protein [(D), cerebellar granule neurons, stained for poly-GA and 4',6'-diamidino-2-phenylindole (DAPI), scale bar 3 μm]. Numerous tissues also show nuclear RNA foci [(E), cerebellar granule neurons, stained for (GGGGCC)_3_ and DAPI, foci are indicated by arrowheads, scale bar 3 μm]

The *C9orf72* expansion is associated with classical Bunina bodies (Fig. 1), and p62 and TDP-43-positive neuronal cytoplasmic inclusion (NCI) and glial cytoplasmic inclusion pathology in the motor cortex and anterior horns of the spinal cord, and with marked loss of motor neurons [16]. Thus, *C9orf72*-related ALS is a TDP-43 proteinopathy, similar to most other subtypes of ALS. However, the repeat expansion cases have additional characteristic pathology in the cerebellum and hippocampus. p62 and ubiquitin-positive but phosphorylated TDP-43-negative NCI have been identified in the 3 layers of the cerebellar cortex, the hippocampus (especially the pyramidal cells), and the neocortex [16, 24, 64] (Fig. 1c). In addition, neuronal intranuclear inclusions were found in the cerebellar granular cells and hippocampal pyramidal cells [65]. The pathology in these extramotor regions, which were not thought to be affected in ALS, were first described in FTD and FTD-ALS [66, 67], and subsequently in ALS [68], before being associated with the *C9orf72* expansion [65].

The NCIs and neuronal intranuclear inclusions, as well as being positive for ubiquitin and p62, also stain for dipeptide repeat proteins (DPRs) (Fig. 1d), which are thought to be generated through repeat-associated non-ATG translation (RAN; see “Protein Toxicity”) [69]. The sense DPRs (poly-GA, poly-GR, and poly-GP) generally co-localize with the p62-positive NCI in the cerebellum and hippocampus. DPRs are also generated from antisense transcripts, although these appear to stain less than the sense DPRs in postmortem material [69–71]. Both sense and antisense DPRs have been identified in motor areas, including spinal motor neurons [71]. However, the relative frequencies of the DPR species in various neuronal populations remains to be definitively determined. Study of *C9orf72* neuropathology has led to a suggestion as to why parkinsonism is more commonly seen in *C9orf72* ALS cases. An increased number of p62-positive and TDP-43-negative NCIs have been found in the substantia nigra of *C9orf72*-related ALS cases, and this is associated with a marked loss of dopaminergic neurons [38].

Finally, all tissues thus far examined in patients carrying *C9orf72* expansions contain nuclear RNA foci transcribed directly from the *C9orf72* expansion in both a sense and an antisense direction (see “RNA Toxicity”) (Fig. 1e) [72–74].

## Function of the C9orf72 Protein

Little is known about the normal role of the C9orf72 protein. The most conserved residues in C9orf72 are distributed throughout the protein, suggesting that it functions as a single block [75]. The use of 6 independent structure prediction tools to examine the primary sequence of *C9orf72* suggested a number of possible functional domains, including a “M16 peptidase”, a “cytochrome bcl”, a “glycohydrolase”, and a “differentially expressed in normal and neoplasia” (DENN) domain [75]. Interestingly, a DENN domain was predicted by 5 of the 6 tools; DENN proteins are Rab-guanosine triphosphate/guanosine diphosphate exchange factors. Moreover, examination of the predicted secondary structure of C9orf72 also revealed significant homology with DENN proteins [75]. This has led to the suggestion that C9orf72 is important in the regulation of Rab activity and thus membrane trafficking, a proposal supported by another study showing that the C9orf72 protein co-localizes in neurons with Rab proteins and membrane vesicles implicated in autophagy and endocytosis [76]. This study also suggested that mutant C9orf72 had an enhanced interaction with Rab7 and Rab11 compared with controls, suggesting that the role of C9orf72 in membrane trafficking may be perturbed in *C9orf72* disease [76]. Alsin, another protein associated with early-onset ALS, is also thought to be a DENN protein with a role in membrane trafficking [77]. One note of caution in interpreting the report of Farg et al. [76] is that a widely accepted C9orf72 antibody with optimal sensitivity and specificity has yet to emerge.

An alternative approach, which is not dependent on an antibody, is to determine locations where the *C9orf72* gene is expressed. Transcription of the mouse *C9orf72* ortholog is enriched in neuronal populations vulnerable to ALS/FTD [78]. This is in direct contrast to other proteins implicated in genetic variants of ALS, including TDP-43 and SOD1, which are ubiquitously expressed. If haploinsufficiency is crucial to the pathophysiology of *C9orf72* disease, then understanding the role of the *C9orf72* proteins in these neurons is likely to be key, whereas if gain-of-toxicity is the mechanism then vulnerable populations may be selected by *C9orf72* expression.

As well as studying C9orf72 protein function directly, study of modifiers of *C9orf72*-disease has the potential to illuminate detail about the normal and the pathological role of the protein. A genome-wide association study identified single nucleotide polymorphisms in TMEM106B as a risk factor for FTD with TDP-43 positive pathology (which includes *C9orf72*-related FTD) [79]. The protein product of TMEM106B is localized to the lysosome. The haplotype associated with higher risk of FTD-TDP, more particularly the major, or T, allele of rs1990622, has recently been investigated in the context of *C9orf72* disease [80]. The major allele is present at a higher than control frequency in patients with *C9orf72* FTD and is associated with an earlier age of onset in *GRN*-related FTD. However, in patients with *C9orf72* FTD, the major allele is associated with a later age of onset and death. This fascinating complexity suggests that both proteins have similar functions and, notably, that membrane trafficking is a component of lysosome function. It has been suggested that the protective isoform of TMEM106B is expressed at a lower level because of increased degradation mediated via altered glycosylation [81]. Interestingly, in contrast to the *C9orf72* FTD findings, it has been shown that neither TMEM106B allele is significantly associated with a *C9orf72*-related ALS presentation [82]. Why the TMEM106B genotype modifies the risk of 1 phenotype and not the other is unknown, but this suggests that the mechanism of neurotoxicity may be different in each case.

Another study aiming to identify genetic modifiers of *C9orf72* disease studied genetic risk factors already associated with ALS in *C9orf72*- expansion carriers and controls. These included altered copy number of survival of motor neuron 1 (*SMN1*) and survival of motor neuron 2 (*SMN2*), CAG repeat expansion of ataxin 2 (*ATXN2*) and GCG repeat expansion of nonimprinted in Prader-Willi/Angelman syndrome 1 (*NIPA1*) [83]. Only *ATXN2* expansions of > 27 units were present at a higher rate in the *C9orf72* expansion carriers, mirroring the findings in ALS more generally [84]. When this result was broken down by phenotype, it was striking that intermediate-length *ATXN2* expansions were present in 2–3 % of patients with ALS or ALS/FTD but were absent in 75 patients with FTD, suggesting that expansion of *ATXN2* may predispose *C9orf72* expansion carriers to develop ALS or ALS/FTD rather than pure FTD. A recent study has confirmed these findings in a larger cohort of patients with ALS, ALS/FTD, and FTD. More than 28 unit expansions of *ATXN2* were present at a higher frequency than in controls in both patients with ALS and ALS/FTD but not patients with pure FTD, and this was true in groups with and without *C9orf72* expansions [85]. It has been suggested that polyglutamine (polyQ) expansion of ATXN2 increases the stability of the protein, enhances its interaction with TDP-43, and may promote cytoplasmic mislocalization of TDP-43 [84]. TDP-43 pathology is a feature of both ALS and FTD, and it remains to be discovered why expansions of *ATXN2* predispose to ALS and not FTD. The fact that this effect appears to be present independently of *C9orf72* expansion suggests that ATXN2 may have an impact on the “final common pathway” of disease.

## Pathogenic Mechanism(s) of the *C9orf72* Repeat Expansion

The mechanism of neurotoxicity in *C9orf72* disease is unknown. However, evidence is being gathered for 3 potential mechanisms, all of which have precedence in other neurological diseases mediated by repeat expansions: 1) RNA-based toxicity of the transcribed repeat; 2) protein-based toxicity via translation of the expanded RNA to form DPRs; and 3) haploinsufficiency.

### RNA Toxicity

**Fig. 2:**
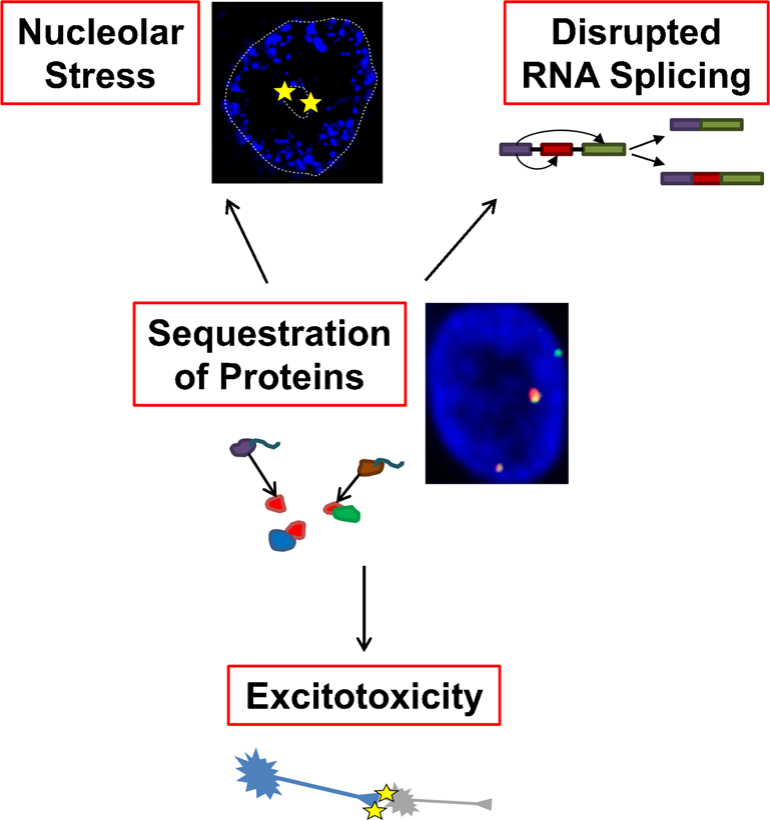
Proposed mechanisms of toxicity mediated by protein sequestration to RNA foci. Various proteins have been shown to be sequestered to RNA foci transcribed either in the sense or antisense direction from the GGGGCC repeat sequence. Proposed mechanisms include sequestration of proteins important to mRNA splicing with consequent disruption of RNA splicing; sequestration of adenosine deaminase, RNA-specific, B2 (ADARB2) (nonfunctional) leading to increased susceptibility to excitotoxicity; and sequestration of nucleolin producing nucleolar stress

RNA foci formed from the repeat sequence were observed in the earliest studies of *C9orf72* disease [9]. The numbers of RNA foci have been correlated with pathogenic severity in cell models [86, 87], and in tissue from FTD cases [73]. A number of molecular phenotypes have been linked to the RNA foci [73, 74, 86–89]. Most groups appear to be exploring the idea that RNA foci sequester and therefore alter the function of certain proteins, including, for example, adenosine deaminase, RNA-specific, B2 leading to excitotoxicity [87], and nucleolin leading to nucleolar stress (Fig. 2) [89]. In view of the relatively late age of onset of *C9orf72* disease and the extremely variable phenotype, we have proposed a model whereby dynamic sequestration of a relatively large number of RNA binding proteins might have a low-level effect on nuclear speckle function (Fig. 2), which, in time, might precipitate disease [72]. Interestingly, we and others, have observed RNA foci in tissue from presymptomatic patients with no clinical disease [72, 73].

It is now clear that stabilized RNA foci are formed from transcription of the GGGGCC repeat in the sense and the antisense direction [70, 73, 74, 89]. Binding partners of the sense and antisense RNA foci are broadly similar and are significantly enriched for RNA-binding proteins [89]. The relative importance of sense and antisense species to the pathophysiology of neuronal injury remains to be determined.

Observations of the transcribed repeat also have a bearing on the formation of DPRs as this requires inappropriate nuclear export to allow the RNA to access the translation machinery. Indeed, cytoplasmic RNA foci have been observed in CNS neuronal populations, including motor neurons, by ourselves and others [72, 74]. In a postmitotic neuron this could not be achieved by nuclear extrusion during mitosis. We have shown that sense RNA foci interact directly with export adaptors, including Aly/REF export factor (ALYREF) [72], and we propose that this interaction might inappropriately license the repeat RNA for export to the cytoplasm. If DPRs are toxic, this represents an attractive therapeutic target. It is interesting to note that pathological analysis suggests that the coincidence in cells of sense/antisense RNA foci and DPR inclusions is relatively low [70], which may suggest that the formation of transcripts into foci and the cytoplasmic export of transcripts for translation, if not mutually exclusive, are mediated independently. If one of these processes is identified as toxic and one as protective then modulating this decision point may represent a therapeutic target.

It is also possible that RNA species transcribed from the hexanucleotide repeat are toxic by some other mechanism. For example, it has been suggested that the sense and antisense transcripts might form inappropriate double-stranded species, which could initiate apoptosis [90]. However, preliminary data suggest that denaturation and renaturation of the GGGGCC repeat in the presence of its antisense complimentary sequence produces a relatively small proportion of double-stranded RNA, in contrast to a similar experiment using the myotonic dystrophy associated CUG repeat [91]. This study concluded that their observations were the result of formation of the GGGGCC repeat sequence into unimolecular stabilized G-quadruplexes where bases are not readily available for Watson–Crick pairing. Study of the secondary structure of the transcribed repeat is in itself likely to be useful in the development of therapies, particularly if successful therapy requires prevention of protein binding to the RNA. More recently, 2 studies have suggested that the sense RNA exists in equilibrium between 2 possible confirmations: a hairpin loop and a G-quadruplex [89, 92]. In contrast, the antisense RNA appears not to be able to adopt a G-quadruplex conformation and to therefore exist primarily in a hairpin loop [89]. These in vitro findings are informative and have already led to the development of a potential small molecule therapeutic agent (see “Therapeutics for C9orf72 Disease”), but should be interpreted with caution—the effect of the normal neuronal environment is unknown.

### Protein Toxicity

Precedence from other repeat expansion disorders led to the search for and the discovery of DPRs in *C9orf72* disease [93]. Antibodies were developed to 3 different proteins, poly-(Gly-Ala), poly-(Gly-Pro), and poly-(Gly-Arg), based on the possible reading frames from which the expansion could be translated. All 3 species were identified within ubiquitinated neuronal cytoplasmic inclusions in *C9orf72* patients. Following the description of antisense transcription of the repeat sequence 2 further proteins were identified corresponding to the 2 additional distinct reading frames, poly-(Ala-Pro) and poly-(Pro-Arg) [71]. Notably, all of the protein species have been identified within ubiquitinated cytoplasmic inclusions and in several cases were observed to co-aggregate [71].

The poly-PR and poly-GP proteins are associated with a potential ATG start codon, but the other species must be translated via a noncanonical mechanism, which is as yet unknown and may mediate translation of all of the proteins. It is proposed that translation may be initiated directly by the repeat expansion, as has previously been observed [94], so-called RAN translation. In this study of spinocerebellar ataxia 8, a neuromuscular disorder caused by a CAG repeat expansion of the ataxin 8 gene, Zu et al. [94] showed that translation of the repeat sequence occurred independently of the presence of an ATG site. As with the *C9orf72* expansion, translation was demonstrated in 3 independent reading frames corresponding to polyQ, polyserine, and polyalanine. Interestingly, the relative mix of these protein species varied depending on the repeat length. They also concluded that the hairpin secondary structure in the RNA is important for RAN translation as a CAA repeat with similar properties, but without the ability to form a hairpin secondary structure, was translated only in the presence of an ATG start codon. This work has already had a significant effect on the study of *C9orf72* disease, and it is likely that it will continue to do so. Work with a transfected GGGGCC repeat sequence has demonstrated length dependence of translation of the poly-PR and poly-GP proteins in a cell model [70].

DPR appears to be toxic in cell and animal models [95–97]. However, neuropathological studies find no relationship between the extent of the DPR pathology and clinical severity [98]. In fact, levels of the aberrantly translated protein appear to correlate inversely with vulnerability of different neuronal groups to neurodegeneration in autopsy material, in direct contrast to the levels of TDP-43-positive inclusions [99]. This may be consistent with a protective role for the formation of DPRs. However, it should be noted that this was a study of pathological material and at the end stage of disease it is impossible to rule out the possibility that the neurons that have already died are the ones containing the highest burden of DPRs. Time course studies in model systems will be required to shed light on this ambiguity.

**Fig. 3:**
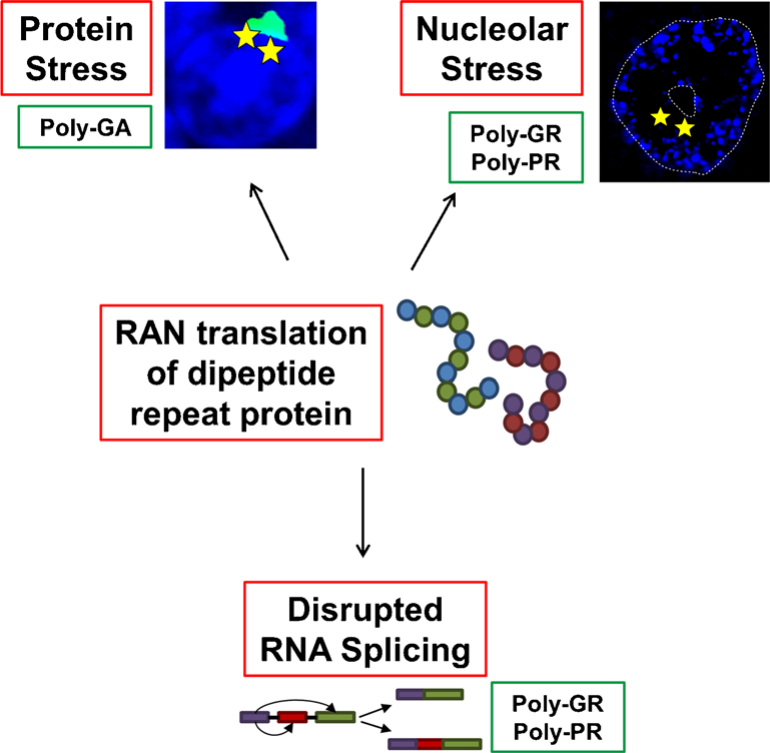
Proposed mechanisms of toxicity mediated via translation of dipeptide repeat protein (DPR). Both sense and antisense, or GGGGCC and GGCCCC repeat RNA species are observed to be translated into DPRs. The absence of traditional ATG start sites has led to the proposal that this occurs via a repeat-associated initial or repeat-associated non-ATG (RAN) translation. The various DPR sequences are proposed to be toxic via a number of mechanisms, including disruption of normal processing of RNA-binding proteins associated with the nucleolus leading to nucleolar stress and/or disruption of normal RNA splicing; or disruption of normal protein processing pathways leading to formation of protein inclusions and toxic protein stress. Implicated DPR species are shown for each mechanism

Very recently, 3 intriguing studies have moved this debate forwards. Kwon et al. [96] have provided evidence from a cell model that the poly-GR and poly-PR DPRs may bind irreversibly to the nucleolus, leading to toxicity via disruption of pre-mRNA splicing and ribosome synthesis (Fig. 3) [96]. The association SR of domains with nucleoli has been observed previously and shown to be dependent on phosphorylation [100]. Kwon et al. [96] have extended this finding to SR domain-containing proteins implicated in ALS. Moreover, they suggest that the poly-GR and poly-PR proteins associate irreversibly with the nucleolus because, unlike SR domains, they lack serine residues and are therefore unable to undergo phosphorylation by CLK1/2 protein kinases. The nucleolar target of arginine-rich domains is unknown, but the authors speculate that the irreversible binding of DPRs might disrupt the normal processing, and therefore the function, of SR proteins in pre-mRNA splicing, and might also disrupt the normal function of the nucleolus in the synthesis of ribosomal RNA. As evidence in support of this hypothesis, they demonstrate changes in the splicing of the excitatory amino acid transporter-2 (EAAT2) and in the production of small nucleolar RNA upon administration of PR_20_. Although narrow, these changes are reminiscent of molecular phenotypes reported previously in ALS [101]. Intriguingly, this offers an alternative mechanism for the nucleolar stress observed by Haeusler et al. [89] described above. This interesting set of observations awaits confirmation and further development. In particular, the effect of dipeptide repeat length is unknown and a direct link to the disease remains to be established. PR_20_ could be generated from a GGGGCC expansion of 20 units, which is within the range found in controls, although the use of supraphysiological levels in the study may have effectively simulated the presence of a larger expansion.

This proposed model was strengthened shortly after by the work of Mizielinska et al. [97], who demonstrated neurodegeneration in a *Drosophila* model upon expression of poly-GP and poly-PR proteins. Toxicity was not present on expression of poly-PA protein and was relatively minor upon expression of poly-GA protein [97]. Importantly, this study included an “RNA-only” model by expression of stop codon-interrupted GGGGCC repeats, which produced RNA foci but not DPRs. The “RNA-only” model formed RNA foci but was not toxic at any repeat length tested (up to 288 repeats). The authors note that the repeat lengths tested are much shorter than expansion lengths present in patients with *C9orf72* disease and therefore this result does not exclude RNA toxicity. They did not describe any length dependence of the DPR toxicity, but they did show amelioration of toxicity by inhibition of protein translation.

Potentially bringing these 2 studies together, May et al. [102] examined the pattern of expression of the 5 dipeptide species in human embryonic kidney 293 cells. The arginine-rich peptides produced dot-like intranuclear inclusions, which may correspond to the nucleolar sequestration proposed by Kwon et al. [96]. May et al. [102] described a potential mechanism of pathogenesis associated with poly-GA repeats (Fig. 3). They reported ubiquitination and toxicity of cytoplasmic poly-GA inclusions associated with interaction between poly-GA and a number of components of the ubiquitin–proteasome system, including Unc119. Unc119 co-localized with poly-GA inclusions in brains of patients with *C9orf72* FTD, and knockdown of unc119 was neurotoxic in a cell model.

This fascinating picture is only likely to become more complicated as understanding of the behavior of DPRs grows. Drawing insights from the polyQ literature, a significant number of interacting partners of polyQ proteins have proven to be genetic modifiers of the disease; as many as 45 % of identified interacting partners modified toxicity in a *Drosophila* model of HD [103]. It is possible that a large number of interactions are important, including but not confined to the nucleolus and components of the ubiquitin–proteasome system.

#### Role of TDP-43

As mentioned previously, *C9orf72*-related ALS is a TDP-43 proteinopathy, in common with most other subtypes of ALS, with the exception of SOD1-related ALS [104]. What is currently unclear is how the mislocalization of TDP-43 from the nucleus to the cytoplasm in all these cases contributes to motor neuronal death. However, evidence suggests that the toxicity is most likely due to dysregulation of RNA processing and protein homeostasis, specifically impairment of the ubiquitin proteasome system, the unfolded protein response, and autophagy, rather than TDP-43 aggregates themselves being toxic [105]. Interestingly, p62, which is an autophagy receptor protein encoded by *SQSTM1*, not only co-localizes with TDP-43 aggregates in the brain and spinal cord of *C9orf72*-related cases, but also with the TDP-43 negative, ubiquitin-positive inclusions, which have been observed to contain DPRs, in the extramotor regions of the brain. Thus, dysregulated protein homeostasis appears to be a common feature of *C9orf72*-related ALS and other subtypes of ALS.

### Haploinsufficiency

Reduced expression of *C9orf72* mRNA has been reported in the presence of the expansion [9]. However, this finding is not consistent [86]. Most recently, study of a newly generated *C9orf72* antibody suggests that there is reduced expression of the *C9orf72* protein in the frontal cortex, but not in the cerebellar cortex of *C9orf72* expansion carriers [106].

It has been demonstrated that small expansions of approximately 50 repeats do not reduce *C9orf72* transcription [107], possibly because smaller expansions do not lead to hypermethylation of a CpG island 5’ to the repeat sequence in the promoter region [108, 109]. If smaller repeat lengths are pathogenic [110, 111], then haploinsufficiency is not the responsible mechanism. Developing this story further, CpG hypermethylation of the *C9orf72* promoter has been shown to correlate with the burden of pathology: the presence of promoter hypermethylation is associated with reduced accumulation of DPRs and RNA foci in the CNS of *C9orf72* patients [112]. Moreover, the same study showed in lymphoblastoid cell lines derived from *C9orf72* expansion carriers that demethylation of the promoter led to increased vulnerability of the cells to oxidative and autophagic stress. While not conclusive, this suggests that reduced expression of expanded *C9orf72* might be protective rather than pathogenic. A number of other mechanisms have been proposed for haploinsufficiency: trimethylation of histones H3 and H4 has been reported in *C9orf72* expansion carriers and linked to increased binding of these histones to the repeat sequence with consequent reduced expression of *C9orf72* [113]. Finally, biochemical analysis has suggested that formation of expanded *C9orf72* DNA and RNA into hybrid R loops may also contribute to abortive transcription [89].

Friedreich’s ataxia is a neuromuscular disorder associated with an intronic repeat expansion in the frataxin gene. In this disease the mutation must be homozygous to be pathogenic and haploinsufficiency has been confirmed at the protein level. Drawing parallels with *C9orf72*, the frataxin repeat expansion has been associated with epigenetic silencing. However, additional mechanisms have been identified: blockage of transcription elongation by DNA repeat secondary structure has been demonstrated [114], and the presence of the repeat expansion has been shown to reduce levels of mature frataxin mRNA via interaction with trans-acting splicing factors [115]. The latter mechanism might explain some of the controversy in the measurement of *C9orf72* mRNA as the diversity of splice variants produced might lead to contrasting results in various quantitative PCR assays, depending on the primers utilized. It remains to be seen whether a similar mechanisms are at play in *C9orf72* disease.

Another observation not consistent with a pathogenic role for haploinsufficiency comes from 2 patients with expansions of both *C9orf72* loci; 1 a homozygote and the other a compound heterozygote [107, 116]. Both cases suffered FTD, but neither phenotype was outside the usual phenotypic spectrum. This is not consistent with a pure haploinsufficiency model, which would predict disease severity in proportion to the number of involved alleles. It remains to be seen whether haploinsufficiency is a disease modifier, but this evaluation may be just around the corner if the newly developed antibodies become widely accepted as sensitive and specific.

## Repeat Length as a Modifier of *C9orf72* ALS

Clearly, the first 2 of the potential pathogenic mechanisms discussed above are gain-of-function toxicities. In a gain-of-function scenario, it would be predicted that disease severity is proportional to repeat length. However, a conclusive relationship between repeat length and disease severity has not yet been demonstrated. Measurement of repeat length initially proved technically challenging as the large GC rich region is not amenable to standard PCR-based sequencing. However, several groups have now optimized Southern hybridization-based techniques [14, 117, 118]. Currently, in a pure ALS group, no aspect of the disease phenotype has been shown to correlate significantly with the length of the expansion, regardless of the tissue tested [118, 119]. In FTD, a direct correlation between repeat size in the frontal cortex and age of onset has been demonstrated, and in the cerebellum a threshold repeat size has been associated with reduced survival [118]. This report also indicated that the repeat length in the cerebellum was shorter than in other CNS areas. It is hypothesized that repeat expansions can increase in size through a human lifetime, resulting in significant somatic heterogeneity [120], and, therefore, perhaps the minimum repeat length in the CNS is more reflective of the germline repeat number. If so, the expansion length in the cerebellum may best represent the repeat length that initiated the disease pathogenesis, and the correlation with age of onset in frontal cortex may simply reflect the patient’s age.

An alternative way to approach this problem is to look for evidence of anticipation in *C9orf72*-related disease, which would be highly suggestive of a relationship between repeat size and disease severity. A series of *C9orf72*-FTD and *C9orf72*-ALS families have been described [26, 121], with 7–10 years of anticipation between generations.

Against a direct relationship between repeat length and disease severity is the description of patients with significant clinical disease but a relatively small repeat lengths of < 30 units [110, 111]. Repeat lengths of 7–24 units are associated with the 9p21 risk haplotype [122]. We have recently described a patient with clinical ALS, an intermediate expansion of 16 GGGGCC repeats and the 9p21 risk haplotype, but without the typical neuropathology associated with *C9orf72* disease, including RNA foci, DPR inclusions, and TDP-43-negative, p62-positive neuronal inclusions in extramotor areas [123]. We suggest that the pathological characterization of this case indicates that this individual actually suffered from non-*C9orf72* ALS and the intermediate length expansion was not sufficient to initiate typical *C9orf72*-mediated neuronal injury. Consistent with this, the frequency of 7–24 repeats, unlike that of longer repeats, is equivalent in patients and controls [122]. That patients and controls with intermediate-length expansions also tend to carry the 9p21 risk haplotype may reflect the suggestion made earlier, that the risk haplotype predisposes the region to expand; however, we suggest that a minimum length, probably much greater than 30 repeats, is necessary to initiate *C9orf72*-neuropathology.

## Therapeutics for *C9orf72* Disease

**Fig. 4:**
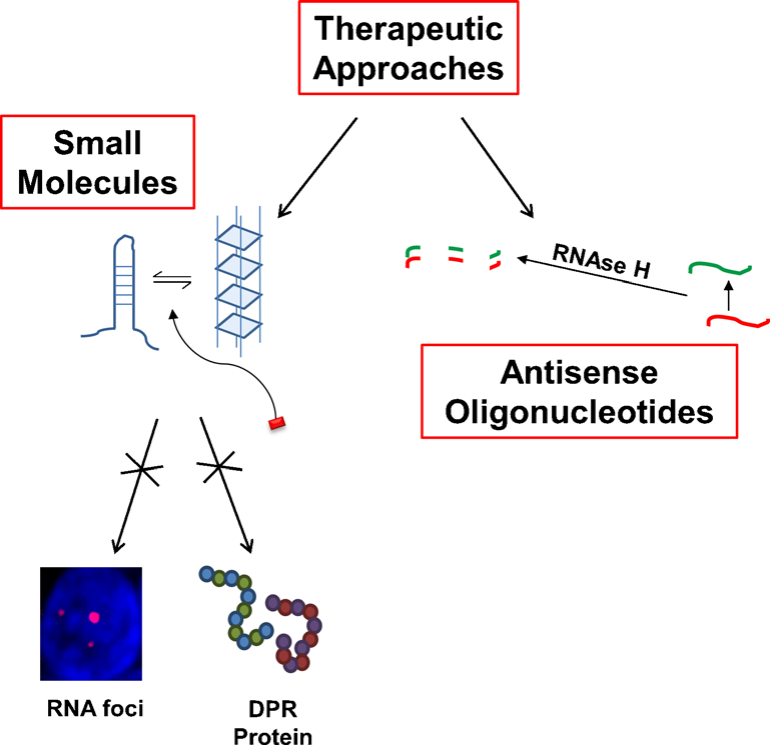
Proposed therapeutic approaches for *C9orf72* disease. At this point, 2 therapeutic approaches have been proposed and partially demonstrated for *C9orf72* disease. First, an antisense oligonucleotide approach whereby the introduced molecule forms a double-stranded complement with the transcribed repeat sequence and thus targets it for degradation by RNAse H. This has been reported to reverse the formation of RNA foci and rescue certain molecular phenotypes. Second, a small molecule approach targeting the secondary structure of GGGGCC repeat RNA, which is thought to be in equilibrium between a hairpin and a G-quadruplex conformation, has been shown to disrupt both formation of RNA foci and translation of dipeptide repeat proteins (DPR)

Understanding of pathogenesis related to *C9orf72* repeat expansion is increasing but some attractive upstream therapeutic targets have already arisen. If disease is initiated by RNA foci and/or DPRs formed from the repeat sequence, then selectively preventing transcription and/or translation of the repeat might prevent toxicity. Some progress has already been made in this and is described in detail elsewhere in this series. However, briefly antisense oligonucleotides have been utilized to promote degradation of *C9orf72* transcripts [73, 86, 87], and more recently the use of small molecules that bind the secondary structure of the repeat sequence have been shown to inhibit formation of RNA foci and translation to form DPRs (Fig. 4) [92].

## Genetic Screening of *C9orf72* in ALS

The discovery of *C9orf72* hexanucleotide repeat expansions as a cause of ALS is hugely important, not least because this genetic subgroup represents such a substantial proportion of patients with ALS. *C9orf72* expansions account for approximately 10–20 % of total ALS cases in different populations, including a proportion of cases with apparently sporadic disease. This frequency means that, for the first time, there is the opportunity to study preclinical through to clinical disease systematically, and also to evaluate a specific molecular subgroup separately when testing new potential neuroprotective agents in clinical trials.

Clinical genetic screening for *C9orf72* expansions can be reliably undertaken by the use of a combination of amplicon-length analysis and repeat primed PCR [124], although Southern blotting is necessary for accurate estimation of the repeat size. Genetic screening in FALS cases is routinely undertaken following counseling of the patients and consent. Given that *C9orf72* expansions are found in up to 7–19 % of patients with ALS with apparently sporadic disease in different populations, the question arises as to whether all ALS cases should be offered screening for changes in this gene. Consensus has not yet been reached in relation to this issue, although genetic counseling considerations for individuals at risk for a *C9orf72* repeat expansion have been reviewed [125, 126]. Psychological, social and ethical implications of genetic testing in ALS are still relatively unexplored.

## Conclusion

Study of *C9orf72* disease is proceeding at a rapid pace and has delivered perhaps the most significant increase in understanding of ALS pathogenesis in the last 20 years. The most recent advances summarized in this review concern the changes in our understanding of potential RNA- and protein-mediated mechanisms of pathogenesis. An interesting convergence has recently occurred where, through different mechanisms, it appears that RNA foci and DPRs may affect the same targets: pre-mRNA splicing mediated by RNA recognition motif (RRM)-containing, SR-containing proteins and the normal function of the nucleolus in ribosome production. The potential involvement of the ribosome is interesting and studies of the “translatome” are awaited.

The recent flurry of evidence for toxicity mediated via DPRs is exciting and is reminiscent of the polyQ proteins produced in other repeat-associated neuromuscular diseases. However, other genetic variants of ALS/FTD, most prominently patients with mutations of *TARDBP*, do not have a means of producing a protein-repeat sequence, despite clinical and pathological similarities to the *C9orf72* disease. What is shared is the potential for RNA-mediated toxicity as TDP-43 is a key RNA processing protein [15], and a number of lines of evidence have pointed to its RNA processing function in the pathogenesis of neuronal injury [127, 128]. As the lessons learned from *SOD1*-related ALS have shown over the last 2 decades [129], it is likely that *C9orf72*-mediated ALS will prove to be multifaceted and complex; this is consistent with the wide variability of the clinical phenotype and disease course in this genetic subgroup of patients.

## Electronic Supplementary Material

Below is the link to the electronic supplementary material.

ESM 1 (PDF 1224 kb)
